# The items in the Chinese version of the Montreal cognitive assessment basic discriminate among different severities of Alzheimer’s disease

**DOI:** 10.1186/s12883-019-1513-1

**Published:** 2019-11-04

**Authors:** Yan-Rong Zhang, Yun-Long Ding, Ke-liang Chen, Yan Liu, Can Wei, Ting-ting Zhai, Wen-Juan Wang, Wan-Li Dong

**Affiliations:** 1 Department of Neurology, The First Affiliated Hospital of Soochow University, Suzhou, 215000 Jiangsu China; 2 Department of Neurology, Jingjiang People’s Hospital, the Seventh Affiliated Hospital of Yangzhou University, Jingjiang, 214500 Jiangsu China; 3 Department of Neurology and Institute of Neurology, Huashan Hospital, Shanghai Medical College, Fudan University, Shanghai, 200040 China; 4 Department of Neurology, the First Affiliated Hospital of Soochow University, 188 Shizi Street, Suzhou, 215006 Jiangsu China

**Keywords:** Mild cognitive impairment, Montreal cognitive assessment, Alzheimer’s disease

## Abstract

**Background:**

To determine whether items of the Chinese version of the Montreal Cognitive Assessment Basic (MoCA-BC) could discriminate among cognitively normal controls (NC), and those with mild cognitive impairment (MCI), mild Alzheimer’s disease (AD), and moderate-severe (AD), as well as their sensitivity and specificity.

**Methods:**

MCI (*n* = 456), mild AD (*n* = 502) and moderate-severe AD (*n* = 102) patients were recruited from the memory clinic, Huashan Hospital, Shanghai, China. NC (*n* = 329) were recruited from health checkup outpatients. Five MoCA-BC item scores were collected in interviews.

**Results:**

The MoCA-BC orientation test had high sensitivity and specificity for discrimination among MCI, mild AD and moderate-severe AD. The delayed recall memory test had high sensitivity and specificity for MCI screening. The verbal fluency test was efficient for detecting MCI and differentiating AD severity.

**Conclusions:**

Various items of the MoCA-BC can identify MCI patients early and identify the severity of dementia.

## Background

Mild cognitive impairment (MCI) is a transitional cognitive state between normal ageing and the early stages of dementia. Alzheimer’s disease (AD) is the most common neurodegenerative disease, and the incidence of AD increases with age. Approximately 10–20% of adults over the age of 65 have MCI, and approximately 10% of these patients may progress to AD [1] . Neuropathology has found that MCI patients also had extracellular amyloid deposition and neurofibrillary tangles in the susceptible brain regions of AD patients (such as the olfactory cortex in the medial temporal lobe) [2] . There is a close relationship between MCI and AD.

The Montreal Cognitive Assessment (MoCA) had been used as a quick evaluation scale to detect MCI in the group of highly educated elderly adults with an average education level of 13 years [3, 4]. Several items in the MoCA incorporated tasks that showed wide differences due to formal education or literacy levels, such as the Trail-Making Test, cube copy, and naming low-familiarity animals [5]. The cutoff points to detect MCI or AD have been demonstrated to be different all over the world [6–9]. The Chinese version of the MoCA-B (MoCA-BC), translated with cultural modifications from the original English version, has shown excellent validity and accuracy in distinguishing between NC, MCI, mild and moderate AD among the Chinese elderly with various levels of education [10, 11].

Compared with the MoCA, the MoCA-BC takes less time (the total number of tests is reduced by 3), and the change in some non-memory cognitive function tests improves the sensitivity for screening and the acceptance of the participants. There are nine items assessing different cognitive domains in MoCA-BC, including language, attention, calculation, orientation, memory, concentration, executive function, conceptual thinking, and visual perception.

There is currently no comparison of the items in the MoCA-BC scale among cognitively normal people, and individuals with MCI, mild AD, and moderate-severe AD. We selects five items in MoCA-BC that scored more than five points in a single item; these tests are the verbal fluency test, visual perception test, immediate memory test, delayed recall memory test and orientation test. Our study may help clinicians to fully understand and distinguish the severity of cognitive impairment.

## Methods

### Participants

A total of 1389 participants aged between 50 and 90 years old comprised four groups: 329 cognitively NC, 456 MCI, 502 mild AD, and 102 moderate-severe AD participants. From May 2015 to March 2018, participants with MCI or AD were recruited from the Huashan Hospital Memory Clinic. Cognitively NC participants were recruited from their caregivers.

### Materials and procedure

The inclusion criteria for all participants were normal or nearly normal eyesight and hearing, no history of alcoholism, drug abuse, head trauma or other serious neuropsychiatric diseases and other disease that would affect their performance to complete all neuropsychological assessments. Participants or their legally authorized caregivers provided informed consent before the commencement of the study. This study had been approved by the ethics committee of Huashan Hospital. All participants underwent complete neurological and neuropsychological assessments, brain imaging and other necessary laboratory tests. The diagnosis decision was made independent of MoCA-BC scores.

The diagnosis of MCI was on the basis of the following criteria [12]: complaints of cognitive impairments; normal activities of daily living or slight impairment in instrumental activities of daily living; objective cognitive impairment (Mini-Mental State Examination; MMSE) score > cutoff(> 17 for illiterate, > 21 for education between 1 and 5, > 24 for education more than 6 years) [13], Clinical Dementia Rating (CDR) score of 0.5 [14], and performance on a set of neuropsychological tests that was 1.5 standard deviations (SDs) or more below the normative mean; and no dementia.

AD diagnosis was made according to the National Institute of Aging and Alzheimer’s Association (NAI-AA) diagnostic criteria [15], which was characterized by clear cognitive decline history, insidious onset of symptoms, and initial and most obvious cognitive deficits in memory, language, visuospatial or executive. Mild AD and moderate-severe AD was based on CDR scores: mild AD had a CDR score of 1; moderate-severe AD had 2 or above [14].

Normal controls must have no cognitive impairment, CDR score of 0, MMSE score at the cutoff value or above, the Hamilton Depression Rating Scale score at 12 or less and the modified Hachinski Ischaemic Scale score at 4 or less in the past 2 weeks.

The following clinical and neuropsychological tests were administered: MMSE, MoCA-BC, CDR, MHIS, and HDRS as mentioned above. Five items from the MoCA-BC were selected for assessment in the present study and included measures of language, orientation, memory (instant recall and delayed recall), and visual perception: 1. Measure of language: Ask the participants to name as many types of fruit as possible in one minute, and record that number to obtain the corresponding score; 2. Measure of orientation: Ask the participants to describe the time and place, with a total score of 6; 3. Measure of instant recall: The tester lists 5 words, then the participants recalls as many as possible. Repeats the list twice, the number of repeated words is recorded with a total score of 10. This task did not contribute to the standard MoCA-BC score; 4. Measure of delayed recall: following the other tests, the participants is asked to recall the 5 words and scores 3 points per word for free recall, 2 points following semantic classification clues, and 1 point for multiple choice recall; 5. Measure of visual perception: Identify the number of items in overlapping graphics in one minute, with a total score of 10.

### Statistical analysis

All analyses were carried out in SPSS version 20 for Windows. Pearson correlations were applied for better understanding of the effect of age and education level on performance on the five items. Demographic characteristics for each diagnostic group are described using means and SDs or frequencies where appropriate. Differences in demographic characteristics and cognitive test performance between diagnostic groups were determined by chi-square tests as well as ANOVA with subsequent Bonferroni corrections, with *P* < 0.05 considered significant. Receiver operating characteristic (ROC) curve analysis for MCI or AD dementia was performed to obtain cutoff values with sensitivity and specificity measures for the scores of the five items. The area under the ROC curve (AUC) was used to compare the diagnostic performance of the five items. The level of significance was set at α = 0.05.

## Results

### Demographic information

Correlation analysis between the five item scores and demographic characteristics were carried out in the control group and are summarized in Table 1. It is suggested that the orientation test had no correlation with age and education level. Verbal fluency, visual perception, immediate recall and delayed recall were all negatively, but weakly, correlated with age (*r* < 0.2). Verbal fluency, visual perception, and immediate recall were associated with education. Therefore, to reduce the impact of education level on the item scores of MoCA-BC, we divided the population into three levels according to the years of education: low level (≤6 years), middle level (7–12 years), and high level (> 12 years). The study analysed the difference in the five sub-test scores in the MoCA-BC between the NC group, MCI group, mild AD group and moderate-severe AD group based on level of education.

**Table 1:** Correlation analysis between item scores and demographic characteristics

Item	Verbal fluency		Orientation		Visual perception		Immediate recall		Delayed recall	
	*r*-value	*P*-value	*r*-value	*P*-value	*r*-value	*P*-value	*r*-value	*P*-value	*r*-value	*P*-value
Age	−0.146	< 0.05	− 0.038	> 0.05	− 0.157	< 0.05	−0.145	< 0.05	− 0.136	< 0.05
Education	0.171	< 0.05	−0.015	> 0.05	0.292	< 0.05	0.158	< 0.05	0.039	> 0.05

The demographic information according to education in the NC group, MCI group, mild AD group and moderate-severe AD group is shown in Table 2, and there was no difference in education between the subgroups (*P* > 0.05). In the low and middle-level education groups, there was no difference in sex ratio among the NC group, MCI group, mild AD group and moderate-severe AD group. In the middle and high-education level groups, there were differences in age among the NC group, MCI group, mild AD group and moderate-severe AD group.

**Table 2:** Comparison of demographic information according to level of education

	Low-level education				
Group	NC (*N* = 11)	MCI (*N* = 26)	Mild AD (*N* = 82)	Moderate-severe AD (*N* = 12)	*P*-Value
Factor					
Education years (X̄±s)	2.4 ± 2.8	3.7 ± 2.4	3.2 ± 2.6	1.5 ± 2.3	0.08
Age (X̄±s)	69.2 ± 10.9	66.3 ± 8.6	70.1 ± 8.3	69.9 ± 8.6	0.288
Sex					0.382
Male	6	11	27	3	
Female	5	15	55	9	
	Middle-level education				
Group	NC (*N* = 39)	MCI (*N* = 131)	Mild AD (*N* = 186)	Moderate-severe AD (*N* = 25)	*P*-value
Factor					
Education years (X̄±s)	9.5 ± 3.1	9.3 ± 2.9	9.6 ± 2.4	8.9 ± 3.1	0.53
Age (X̄±s)	65.6 ± 9.0	65.5 ± 7.8	68.3 ± 9.0	67.4 ± 10.6	0.03
Sex					0.389
Male	17	54	85	7	
Female	22	77	101	18	
	High-level education				
Group	NC (*N* = 279)	MCI (*N* = 299)	Mild AD (*N* = 234)	Moderate-severe AD(*N* = 65)	*P*-value
Factor					
Education years (X̄±s)	11.8 ± 3.0	12.3 ± 3.4	12.0 ± 6.2	11.6 ± 3.2	0.56
Age (X̄±s)	65.9 ± 8.2	69.6 ± 8.1	70.8 ± 9.1	69.4 ± 9.7	0
Sex					0
Male	92	152	121	28	
Female	187	147	113	37	

NC, cognitively normal controls; MCI, mild cognitive impairment; AD, Alzheimer’s disease

### Comparison of five items among NC, MCI, mild and moderate AD

We show the five item scores (verbal fluency, orientation, visual perception, immediate recall and delayed recall) for each subgroup in Table 3. There were significant differences among subgroup means for the five item scores, and the significance was less than 0.05. After post hoc pairwise between-group comparisons, we found that the verbal fluency test scores were significantly different between each diagnostic subgroup. There was no difference between the NC and MCI groups in the orientation test, but there were differences among the MCI, mild AD, and moderate-severe AD groups. Visual perception and immediate recall tests differed among each diagnostic subgroup at the high-education level, while at the low-education level there was no difference between the NC and MCI groups. The delayed recall test did not differ between mild AD and moderate-severe AD groups, but there was a difference between the NC, MCI, and mild AD groups.

**Table 3:** Comparison of the five items among 4 groups (X̄±s)

	Index (*X̄±s*)	NC	MCI	Mild AD	Moderate-severe AD	*F* (*P*)	*Bonferroni correction*
Low-level education (n = 131)	Verbal fluency	11.1 ± 2.7	8.2 ± 2.5	6.3 ± 2.3	4.3 ± 1.7	21.743 (0.000)	a; b; c; d; e; f;
	Orientation	6 ± 0	5.7 ± 0.5	3.7 ± 1.7	1.4 ± 0.8	36.481 (0.000)	b; c; d; e; f;
	Visual perception	5.9 ± 2.6	4.1 ± 2.4	2.6 ± 2.3	1.1 ± 1.3	12.022 (0.000)	b; c; d; e;
	Immediate recall	7.8 ± 1.7	6.8 ± 2.5	5.1 ± 2.4	2.6 ± 2.1	13.419 (0.000)	b; c; d; e; f;
	Delayed recall	9.4 ± 3.1	5.2 ± 3.4	1.9 ± 2.8	1 ± 1.9	28.411 (0.000)	a; b; c; d; e;
Middle-level education (*n* = 381)	Verbal fluency	10.8 ± 2.2	9.5 ± 2.7	7.3 ± 2.5	4.2 ± 2.7	53.341 (0.000)	a; b; c; d; e; f;
	Orientation	5.9 ± 0.2	5.7 ± 0.5	4.2 ± 1.3	2.2 ± 1.2	115.913 (0.000)	b; c; d; e; f;
	Visual perception	7.3 ± 1.6	6.3 ± 2.3	4.6 ± 2.5	2.3 ± 1.9	37.701 (0.000)	b; c; d; e; f;
	Immediate recall	8.1 ± 1.3	7.2 ± 1.8	6.1 ± 2.1	3.8 ± 2.5	34.823 (0.000)	b; c; d; e; f;
	Delayed recall	10.0 ± 2.5	6.5 ± 3.5	3.3 ± 3.1	1.6 ± 2.2	70.979 (0.000)	a; b; c; d; e;
High-level education (*n* = 877)	Verbal fluency	11.3 ± 2.8	9.2 ± 2.5	6.9 ± 2.4	5.4 ± 2.3	167.674 (0.000)	a; b; c; d; e; f;
	Orientation	5.9 ± 0.3	5.7 ± 0.5	4.2 ± 1.4	2.8 ± 1.3	347.124 (0.000)	b; c; d; e; f;
	Visual perception	7.5 ± 1.9	6.2 ± 2.3	4.1 ± 2.6	2.9 ± 2.7	130.8 (0.000)	a; b; c; d; e; f;
	Immediate recall	8.4 ± 1.4	7.4 ± 1.7	6.1 ± 2.2	5.1 ± 2.2	96.481 (0.000)	a; b; c; d; e; f;
	Delayed recall	10.2 ± 3.4	5.5 ± 3.7	2.3 ± 3.1	1.1 ± 1.8	284.412 (0.000)	a; b; c; d; e;

a: *p* < 0.05,NC vs. MCI; b: *p* < 0.05, NC vs. mild AD; c: *p* < 0.05, NC vs. moderate-severe AD; d: *p* < 0.05, MCI vs. mild AD; e: *p* < 0.05, MCI vs. moderate AD; f: *p* < 0.05, mild AD vs. moderate AD. NC, cognitively normal controls; MCI, mild cognitive impairment; AD, Alzheimer’s disease

### ROC analysis of the five items between controls and individuals with MCI and AD

**Fig. 1:**
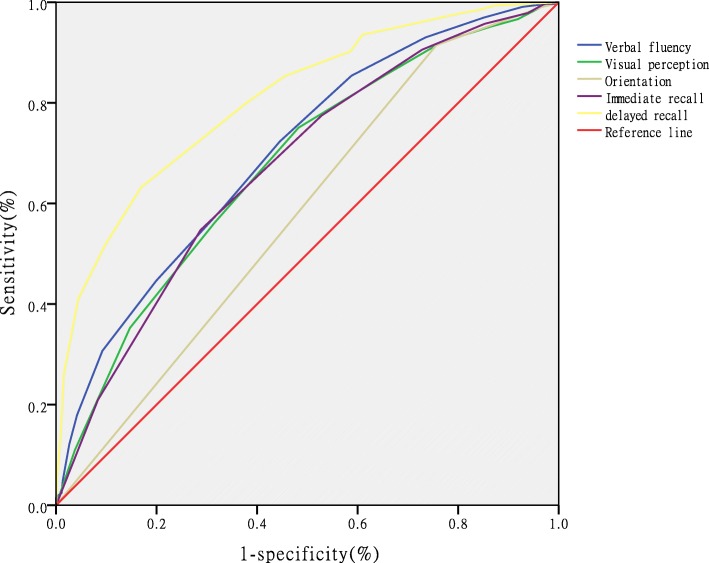
Receiver operating characteristic curve analysis of the five items for distinguishing MCI from NC

**Fig. 2:**
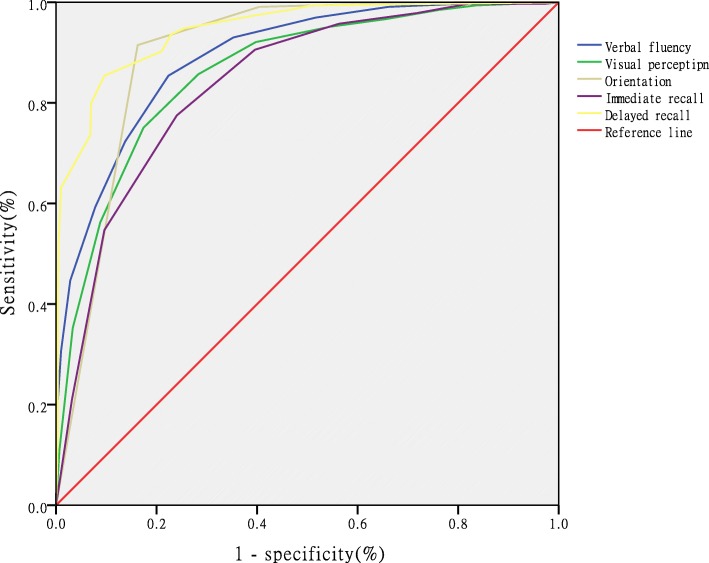
Receiver operating characteristic curve analysis of the five items for distinguishing AD from NC

An ROC analysis of each item was performed for discriminating those with MCI from NC, and the delayed recall item with an AUC of 0.808 (see Table 4, Fig. 1) showed excellent sensitivity and specificity. The optimal cutoff score of each item was ranked in Table 4. Each item for the detection of AD had a higher AUC (0.945) than for the detection of MCI, and the most effective item was the delayed recall test (Table 5, Fig. 2).

**Table 4:** Sensitivity and specificity of the five items for distinguishing MCI from NC

Item	AUC	95% *CI*	Cutoff	Sensitivity %	Specificity %
Verbal fluency	0.701	0.664–0.737	≤9	72.3	55.4
Orientation	0.675	0.637–0.713	≤5	91.5	24.3
Visual perception	0.579	0.539–0.619	≤7	75.1	51.7
Immediate recall	0.673	0.635–0.711	≤8	54.7	71.2
Delayed recall	0.808	0.777–0.839	≤9	63.2	83.1

AUC: area under curve; CI: confidence interval

**Table 5:** Sensitivity and specificity of 5 items for distinguishing AD from NC

Item	AUC	95% *CI*	Cutoff	Sensitivity %	Specificity %
Verbal fluency	0.892	0.872–0.913	≤9	85.4	77.6
Orientation	0.862	0.838–0.887	≤5	91.5	83.8
Visual perception	0.899	0.879–0.919	≤6	75.1	82.6
Immediate recall	0.840	0.814–0.866	≤7	77.5	76
Delayed recall	0.945	0.913–0.959	≤4	93.6	77.2

AUC: area under curve; CI: confidence interval

## Discussion

The purpose of this study was to analyse the validity of the five MoCA-BC items (verbal fluency, orientation, visual perception, immediate recall, and delayed recall) for screening cognitive impairment and the severity of cognitive impairment. The optimal cutoff of the five items in screening for MCI and AD was analysed, which is helpful for clinicians to more intuitively judge these five domains of cognitive impairment. We found that there were differences in age and gender among the four groups in the high-education level groups, and there was an extremely weak negative correlation between age and cognitive impairment, which is consistent with AD itself being an age-related disease. We used analysis of covariance in the high education level population, using age as a covariate to analyze and control the effect of age on the test results. After that, there were differences between the four groups. In China’s epidemiological survey, women’s cognitive impairment is slightly higher than that of men [13], and studies using multivariate analysis of age groups show that this difference is associated with lower education and chronic disease rates among women [16]. Highly related, it is considered that gender has no significant effect on cognitive test scores. The level of education is considered to be the most important factor in cognitive testing (such as the MoCA and MMSE). The optimal cutoff scores for MCI or AD screening for cognitive impairment in China are different according to the level of education. Therefore, to reduce the influence of cultural bias on scores, this paper analyses the difference of the five item scores between NC, MCI, mild AD and severe-moderate AD according the educational level.

In our study, we found that among the NC, MCI, mild AD, and moderate-severe AD groups, there were differences in delayed recall, verbal fluency, visual perception, immediate recall, and orientation. These relations are similar to F. Clement’s research [17, 18] . Education had no effect on orientation and delayed recall. Our results showed that the orientation test was effective in identifying MCI, mild AD, and moderate to severe AD, but poor discrimination between MCI and NC. Memory impairment was the first impaired domain in MCI individuals who had less disruption in the orientation test [12, 19] . The orientation test was easy to complete and showed a ceiling effect when screening for NC and MCI. The delayed recall test has good validity in identifying NC, MCI, and mild AD groups, but no validity in distinguishing the severity of AD; this is probably owing to a floor effect, because in confirmed AD individuals, delayed recall memory impairment is too serious to complete the delayed recall test. However, when screening for MCI or AD, the AUC for the delayed recall test was the largest, which is more conducive to the early recognition of cognitive dysfunction in MCI or AD patients. This is similar to the previous studies by Lin Huang [11] et al. The memory index score of the MoCA-BC had high sensitivity and specificity for MCI screening, while the non-memory index score of the MoCA-BC had similar effectiveness for discrimination among MCI, mild AD and moderate AD groups.

Our results showed that the verbal fluency test had validity not only in the MCI group but also in the mild and moderate-severe AD groups in the three education subgroups. Visual perception and immediate recall tests had validity for discrimination among the NC, MCI, mild AD, and moderate-severe AD groups only in the high-level education population, while in low- and middle-level education populations the two items had poor validity for screening those with MCI from NC. This may be due to the need for a high level of education to complete the two items and the lower impairment in visual perception and immediate recall in MCI individuals.

Cognitive impairment can be manifested in many aspects, such as memory, orientation, language, executive function, visual perception, calculation, attention, and information processing. The MoCA-BC is a quick, simple, and feasible assessment tool not only for doctors but also for outpatients. Each item in the MoCA-BC is independent, which reflects the characteristics of cognitive impairment of dementia in different stages. Sometimes, elderly people may have vision or hearing impairments, and only part of the test can be completed; therefore, a total score cannot be obtained. The MoCA-BC item score is a good complement for these people. The analysis of the differences between the item scores across groups with different levels of cognitive function is very helpful in distinguishing the types of cognitive dysfunction and determining the field of cognitive impairment. Through each item, we can observe the main areas of cognitive impairment and help identify the types of cognitive impairment, further distinguishing between the recognition of AD, Lewy body dementia, vascular dementia or depression.

Of course, the items of the MoCA-BC scale cannot fully satisfy the identification for the stage of AD, so the MoCA-BC needs to be complemented by a combination of more complicated neuropsychological tests.

## Conclusion

In summary, the MoCA-BC is a comprehensive test that combines sub-items to identify early MCI patients and to identify the severity of dementia. This study observed differences in verbal fluency, visual perception, immediate memory, delayed memory, and orientation among the NC group, MCI group, mild AD group, and moderate-severe AD group. The results of the present study indicate that there is a need for further research on the difference in the item scores between those with AD, Lewy body dementia, vascular dementia and depression.

## Data Availability

The datasets used during the current study are available from the corresponding author on reasonable request.

## Abbreviations

AD: Alzheimer’s disease
AUC: area under the ROC curve (AUC)
MCI: mild cognitive impairment
MMSE: Mini-Mental State Examination
MoCA-BC: the Chinese version of Montreal Cognitive Assessment Basic
NC: normal controls
ROC: Receiver operating characteristic
